# PiggyBac-mediated transgenesis and CRISPR–Cas9 knockout in the greater wax moth, *Galleria mellonella*

**DOI:** 10.1038/s41684-025-01665-7

**Published:** 2026-02-10

**Authors:** James C. Pearce, Jennie S. Campbell, Joann L. Prior, Richard W. Titball, James G. Wakefield

**Affiliations:** 1 Living Systems Institute, University of Exeter, Exeter, UK; 2 Department of Biosciences, Geoffrey Pope Building, University of Exeter, Exeter, UK; 3 Defence Science and Technology Laboratories, Porton Down, UK

**Keywords:** CRISPR-Cas9 genome editing, Transposition, Entomology

## Abstract

The larvae of the greater wax moth, *Galleria mellonella*, are gaining prominence as a versatile nonmammalian in vivo model to study host–pathogen interactions. Their ability to be maintained at 37 °C, coupled with a broad susceptibility to human pathogens and a distinct melanization response that serves as a visual indicator for larval health, positions *G. mellonella* as a powerful resource for infection research. Despite these advantages, the lack of genetic tools, such as those available for zebrafish and *Drosophila melanogaster*, has hindered development of the full potential of *G. mellonella* as a model organism. Here we describe a robust methodology for generating transgenic *G. mellonella* using the PiggyBac transposon system and for precise gene knockouts via CRISPR–Cas9 technology. These advances significantly enhance the utility of *G. mellonella* in molecular research, paving the way for its widespread use as an inexpensive and ethically compatible animal model in infection biology and beyond.

## Abstract (web summary)

The authors used the PiggyBac and CRISPR–Cas9 systems to generate transgenic lines and gene knockouts in the greater wax moth, *Galleria mellonella*. These approaches are expected to broaden the range of applications for this model in preclinical research.

## Main

The larval stage of the greater wax moth, *Galleria mellonella*, is increasingly recognized as a valuable in vivo mammalian replacement model, particularly in the fields of infection, immunology and inflammation^1–9^. They undergo melanization in response to immune challenges and have broad susceptibility to a range of medically important microbes (reviewed in refs. ^1–3,5,10,11^). Their capacity to be maintained at 37 °C confers a considerable advantage over other model systems such as fruit flies or zebrafish, particularly for studies involving human pathogens. Moreover, unlike vertebrate models, *G. mellonella* larvae are not subject to stringent regulatory or licensing requirements. Finally, recent discoveries, such as their unique ability to metabolize polyethylene and polystyrene, independent of their microbiota^12–15^, could yield advances in our understanding of plastic degradation and solutions to plastic waste, underscoring the potential for broad application of *G. mellonella* larvae in research settings.

The availability of multiple *G. mellonella* genomes, first published in 2018^13,16–18^, have resulted in an expanded set of molecular and cellular tools, alongside transcriptomic and proteomic datasets^19–28^. These resources have considerably increased the potential of *G. mellonella* to be developed as an alternative to mammalian infection models. However, the absence of robust genetic manipulation techniques—critical for the insertion, deletion and engineering of genes—remains a limiting factor. Although such techniques have been widely applied to other insects, transgenic and genetically modified approaches in *G. mellonella* have not yet been developed.

Among the most widely adopted tools for genetic modification are the PiggyBac transposase system and CRISPR–Cas9, both of which offer powerful, complementary means of creating transgenic organisms and targeted gene knockouts. The PiggyBac transposase system, isolated from *Trichoplusia ni*^29^, enables the seamless integration of genetic material into TTAA nucleotide sequences across a wide array of animal species, facilitated by a separately provided transposase enzyme^30,31^. The ability of this method to insert large genetic cargos^32^ without the need for specific landing sites, while advantageous, also poses challenges, including variable integration efficiency^33^ and the potential for disruption of endogenous gene function if the insertion occurs within a coding region. CRISPR–Cas9-mediated mutagenesis has rapidly become the gold standard for targeted genetic modification, surpassing other techniques such as zinc finger nucleases and transcription activator-like effector nucleases^34–36^. This technique uses a bipartite type II CRISPR system to direct a CRISPR-associated nuclease (Cas) to specific genomic loci via an RNA guide with a complementary base sequence^37–39^. The resultant double-strand breaks are repaired with varying fidelity by different DNA repair pathways, enabling either the disruption of endogenous gene function or the insertion of exogenous genetic sequences^40^.

In this study, we successfully apply both the PiggyBac and CRISPR–Cas9 systems to *G. mellonella*, demonstrating their efficacy in generating transgenic lines and gene knockouts. These advances considerably enhance the genetic tractability of *G. mellonella*, establishing a foundation for its broader application across diverse research domains.

## Results

### Embryonic development timings indicate a 6-h window for microinjection

**Fig. 1:**
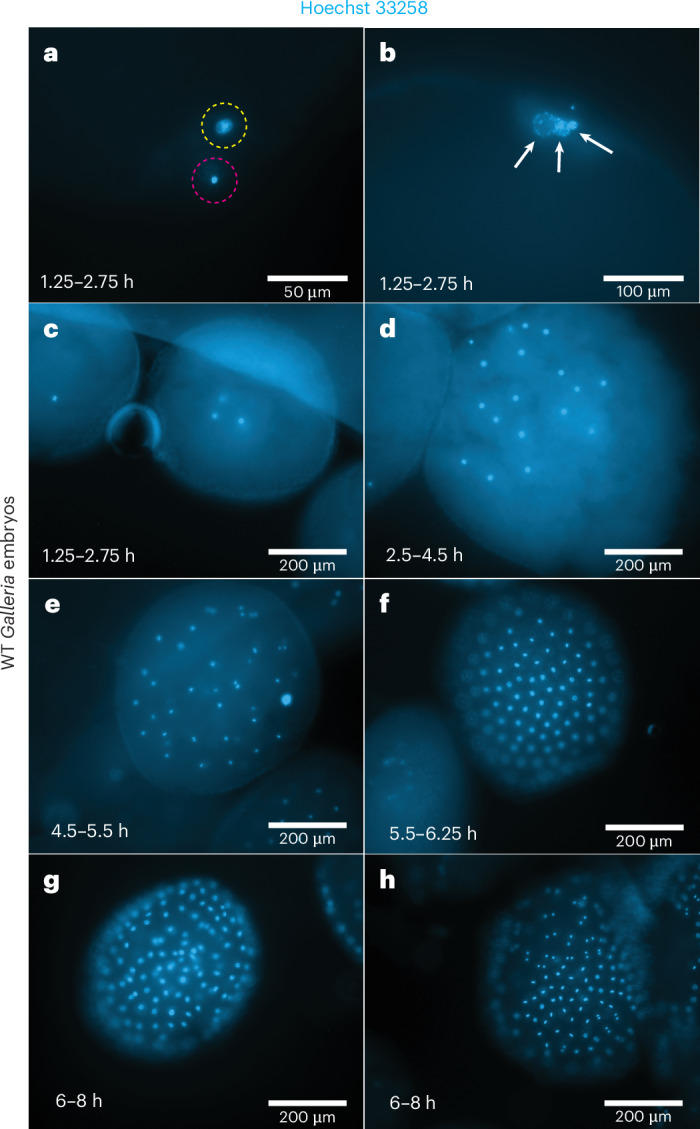
*Galleria mellonella* embryos fixed during set points in development and stained with Hoechst 33258 DNA dye. **a**–**c**, In the first 1.25–2.75 h PO, the sperm nuclei (purple circle) can be seen moving toward the ovum nuclei (yellow circle) (**a**); and nuclei resembling polar bodies gather toward the periphery of the embryo (white arrows) (**b**). This timeframe appears to cover up to the second mitotic division (**c**). **d**,**e**, From 2.5 h to 5.5 h PO, energids migrate toward the periphery with a bias to the anterior pole (**d**); and by 4.5–5.5 h PO, the first ones have just reached the periphery (**e**). Nuclei are dividing synchronously at this point as they share a common cytoplasm. **f**, All energids have reached the periphery by 6.25 h and are still dividing together. **g**,**h**, However, synchronicity begins to be lost from 6 h to 8 h, potentially indicating the onset of cellularization. In **g**, all nuclei appear to be coming out of telophase; however, in **h**, nuclei appear in metaphase, telophase and interphase, with some showing decondensed chromatin.

*Galleria mellonella* were reared at 30 °C and embryos were deposited on egg papers, followed by manual collection and subsequent fixation. Imaging of early embryos, stained for DNA, revealed developmental timings post oviposition (PO). In embryos aged 1.25–2.75 h PO, sperm nuclei could be observed in transit toward the ova nucleus, with polar bodies migrating toward the boundary of the embryo (Fig. 1a–c). A small proportion of embryos in this time window were also observed to have undergone their first mitotic division, with some beginning their second.

In batches of embryos collected within the next 3.5 h (2.75–6.25 h PO), increased numbers of nuclei, migrating toward the periphery of the embryo, were observed (Fig. 1d–f). The nuclei within an individual embryo seemed to be at the same cell cycle stage, as determined by chromosome condensation, alignment and segregation, indicating that, at this time point, they still share a common cytoplasm (Fig. 1d–f). This synchronicity was lost, however, in batches of embryos fixed and imaged 8 h PO, indicating that cellularization occurs between 6.25 h and 8.00 h (Fig. 1g,h). As embryos developed further, a difference in nuclei spacing became noticeable, with the future embryonic tissue being more densely nucleated by 14 h (Supplementary Fig. 1). Together, this analysis provides a time window for injection to generate stable germline transformants within 0–6 h of development following oviposition at 30 °C.

### p*Bm*hsp90:hyPBase is suitable as a donor plasmid for PiggyBac mutagenesis in *G. mellonella*

To inject exogenous material definitively before cellularization, we performed manipulations on 0–2-h-old dechorionated embryos (Methods). Perhaps unsurprisingly, injection with piggyBac DNA plasmids caused substantial mortality (Table 1), with only 6–19% of injected embryos from the five different experiments surviving to pupation. Hatch rates were much lower for injection with piggyBac DNA plasmids than injections with only injection buffer (27.2% for plasmid DNA versus 69.0% for injection buffer only)^41^. However, given the large number of embryos that can be collected and injected within a few hours, we proceeded with this methodology to screen for transformants.

**Table 1:** Hatch and pupation rates for different injection mixes

Constructs	Injection concentration	Strain injected	Number injected	Number hatched	Number pupated	Positive G1 broods
p*Bm*hsp90:GFP/3xP3:DsRed + pH*Bm*A3:PIG	200 ng/μl 200 ng/μl	WT	4,692	1,323	641	0
p*Bm*hsp90:GFP/3xP3:DsRed + pH*Bm*A3:PIG	400 ng/μl 200 ng/μl	WT	400	Not done	51	0
p*Bm*hsp90:GFP/3xP3:DsRed + p*Bm*hsp90:hpPBase	400 ng/μl 200 ng/μl	WT	400	Not done	83	1
p*Gm*hsp90:GFP-αtub1b + p*Bm*hsp90:hpPBase	500 ng/μl 200 ng/μl	WT	1,450	372	283	2
p*Bm*hsp90:histone2AV-MCh/3xP3:DsRed + p*Bm*hsp90:hpPBase	500 ng/μl 200 ng/μl	WT	1,280	327	77	1
GFP-sgRNA + Cas9-MCh protein	2,000 ng/μl ~800 ng/μl	*Bm*hsp90:GFP/ 3xP3:DsRed	400	122	28	14

Hatch rates were calculated by counting unhatched embryos remaining on the slide and subtracting this number from the total injected. Pupation rates were determined by the number of larvae that entered pupation; however, not all G0 pupae emerged as adults or were fertile. Where hatch rates were not determined, ‘Not done’ has been recorded.

Initially, we injected a 200:200 ng/μl plasmid mix consisting of p*Bm*hsp90:GFP/3xP3:DsRed donor and pHA3PIG (*Bombyx mori* actin-3 driven) helper, as used by Tsubota et al.^42^. This plasmid was expected to drive green fluorescent protein (GFP) ubiquitously (under the control of the *hsp90* promoter) and dsRed in the developing eye (under the control of the P3 promoter). However, we did not observe any GFP or dsRed fluorescence in any G0 larvae or G1 *G. mellonella*, despite the large number of embryonic injections (*N* = 4,692) and G1 broods screened (*N* > 600) (Table 1). This finding indicated either that this helper plasmid had very low activity in *G. mellonella*, due to inactivity of the unmodified transposase or incompatibility of the *B. mori* actin-3 promoter, or (in what was considered a less likely scenario) that both reporter constructs within the donor expression cassette were unable to efficiently drive fluorescent protein expression.

**Fig. 2:**
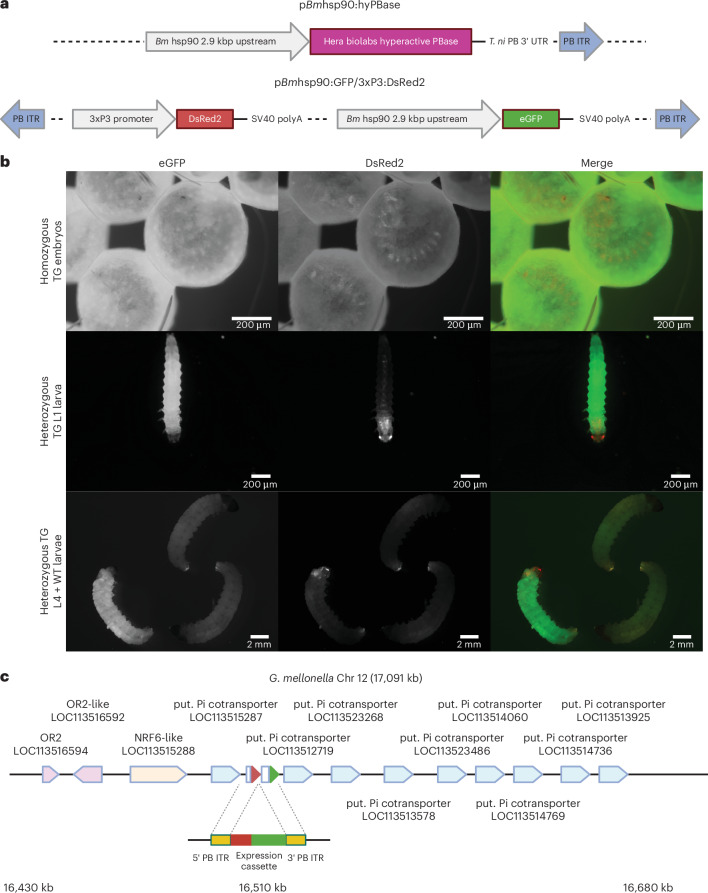
Generation of the first transgenic *G. mellonella* reporter line expressing both GFP and DsRed. **a**, The helper and donor constructs used to develop the *Bm*hsp90:GFP/3xP3:DsRed transgenic strain are represented. **b**, Both eGFP and DsRed expression can be observed in the late embryo (top row; G4 animals), first instar larvae (middle row; G2 animals) and fourth instar larvae (bottom row; G4 animals). eGFP expression is very bright within the unmelanized tissues, while DsRed expression is limited to neural tissue including the brain, optic nerves and stemmata. **c**, The transgenic cassette appears to be inserted within an inorganic phosphate cotransporter gene cluster on chromosome 12 in the intergenic space between LOC113515287 and LOC113512789, adjacent to a small cluster of putative (put.) odorant-like receptors.

To address this issue, we investigated whether an alternative promoter–transposase helper construct could increase transformation efficiency sufficiently to generate a transgenic line. Two sets of injections were performed with the same donor plasmid, p*Bm*hsp90:GFP/3xP3:DsRed, but at a higher concentration (400 ng/μl), either with 200 ng/μl of the original pHA3PIG helper or with a different helper plasmid, p*Bm*hsp90:hyPBase, which encodes a hyperactive PiggyBac transposase mutant, driven by the same *Bombyx* hsp90 upstream sequence as used in the donor^42^ (Fig. 2a). Although the hyperactive transposase used was codon optimized for expression in mammalian rather than insect systems, Eckermann and colleagues had previously found no significant difference in transformation efficiency between insect and mammalian codon-optimized hyperactive transposases in multiple insect species^43^. Mosaic green fluorescence was observed in several G0 larvae from the p*Bm*hsp90:hyPBase injection group, while none was observed in the pHA3:PIG group (Table 1). From the p*Bm*hsp90:hyPBase group, eGFP- and DsRed-positive transgenic larvae were recovered from the brood of a single G0 adult–wild-type (WT) cross (Table 1). Although the promoters differed and direct comparisons cannot be drawn, the p*Bm*hsp90:hyPBase plasmid might be a more suitable helper in this species.

### The *B. mori* hsp90 promoter sequence drives strong but not constitutive activity in *G. mellonella*, while the 3xP3 promoter might be neural specific

In larvae transformed with p*Bm*hsp90:GFP/3xP3:DsRed, bright eGFP expression could be observed in larval, pupal and adult stages. However, instead of ubiquitous expression, eGFP expression in embryos appeared to be limited to the vitellophages and was absent both from the germ band and developing nascent larva until just before eclosion (Fig. 2b). In larvae, eGFP expression was strongest in the muscle, fat body and Malpighian tubules with weaker expression seen in the gut, silk glands and epidermis.

As expected, DsRed expression was observed to be present in, and solely within, neural tissue, with the strongest expression in the eyes, optic nerves, brain and segmental ganglia (Fig. 2b). It was first observed at around day 5 of embryonic development at 30 °C, at the anterior and in a segmented pattern within the embryo, perhaps corresponding to the developing eyes and segmental neural centers.

To map the insertion site in one of these transformants, inverse polymerase chain reaction (PCR) was undertaken. The flanking regions of the *Bm*hsp90:GFP/3xP3:DsRed expression cassette were found to correspond to the proximal end of chromosome 12, within an intergenic region between LOC1131515287 and LOC1131512719, in a cluster of putative inorganic phosphate cotransporters (Fig. 2c and Supplementary Fig. 2). Visual monitoring of outward health and developmental timings for this transgenic line, across multiple generations, showed no difference to WT, suggesting that transgene expression at this locus is not deleterious to the organism.

### Generation of *G. mellonella* α-tubulin and histone cellular reporter lines

**Fig. 3:**
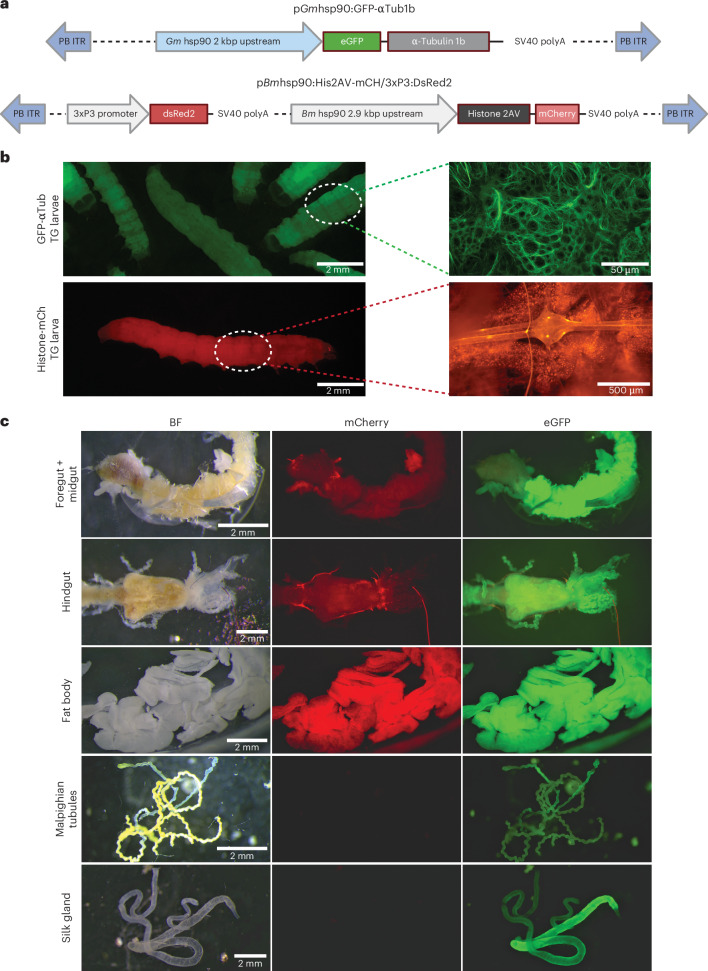
Generation of GFP-tubulin and histone-mCherry reporter lines. **a**, Constructs used to generate transgenic lines *Gm*hsp90:GFP-αtub1b and *Bm*hsp90:his2av-mCh. **b**, *Gm*hsp90:GFP-αtub1b larvae (top left) with fat body tissue fixed and stained with an anti-GFP antibody (top right); and *Bm*hsp90:his2av-mCh (bottom left) larvae with fat body and dorsal neural ganglion imaged live (bottom right). Zoomed panels are not images from the same larvae. Expected cytoskeletal distribution of eGFP was observed, corresponding with expected localization of tubulin, while a nuclear localization was observed for mCherry. **c**, Brightfield (BF), mCherry and eGFP tissue expression patterns in a strain with both *Bm*hsp90:his2av-mCh/*Gm*hsp90:GFP-αtub1b expression cassettes. Strong fat body expression was observed for both fluorophores, with the *G. mellonella hsp90* promoter that seemed to drive strongest expression in gut and silk gland, while the *B. mori hsp90* promoter was stronger in epidermal and muscle tissue (not shown), but very weak in silk glands and Malpighian tubules.

To investigate whether a *G. mellonella hsp90* promoter might drive constitutive expression, and whether PiggyBac would be suitable for creating reporters of cellular and subcellular dynamics, two DNA constructs were generated. In the first construct (*Gm*hsp90:GFP-αtub1b), the *G. mellonella* α-tubulin 1b gene—one of several α tubulin genes in the *G. mellonella* genome—was N-terminally tagged with eGFP and placed downstream of a 2-kb region corresponding to the *G. mellonella hsp90* promoter (Fig. 3a). In the second construct, a 2-kb region corresponding to the *B. mori hsp90* promoter was placed upstream of the *G. mellonella* monocistronic histone 2A variant, with a C-terminal mCherry tag (*Bm*hsp90:his2av-mCh) (Fig. 3a).

Hatch rates for injected embryos were similar for both constructs, at around 26% survival (Table 1). However, while 20% of the 1,450 *Gm*hsp90:GFP-αtub1b injected embryos reached pupation, only 6% of the 1,280 embryos injected with *Bm*hsp90:his2av-mCh reached this stage (Table 1). This result may indicate additional toxicity associated with the integration of the histone expression cassette, either due to overexpression of this histone variant or the specific insertion locus.

Transgenic lines for both constructs were obtained. Although inverse PCR failed to locate the insertion locus for either line, fluorescence corresponding to microtubules and nuclei, respectively, could be observed in embryos (not shown) and larvae (Fig. 3b). Interestingly, we found differences between the expression pattern of the *B. mori* and *G. mellonella hsp90*-integrated lines. Strong eGFP expression for the *G. mellonella* hsp90-GFP-αtub1b construct was observed in the fat body, hindgut, midgut and silk glands, with somewhat weaker expression in the Malpighian tubules (Fig. 3c), as well as the muscle and epidermis (not shown). By contrast, the *B. mori* *hsp90* promoter drove strong expression of mCherry-His2Av in the fat body, weaker expression in the gut, and appeared absent or present at very low levels in the silk glands and Malpighian tubules (Fig. 3c). This finding is in contrast with results previously observed for eGFP expression in the *Bm*hsp90:GFP/3xP3:DsRed strain, where very strong and moderate expression was observed in Malpighian tubules and silk gland, respectively, suggesting either insertion-specific differences or selective suppression of *His2av* expression in those tissues. Embryonic expression was weak for both promoters, although some germline activity could be observed for the *G. mellonella* *hsp90* promoter, in contrast to the vitellophages in the *B. mori* *hsp90* line (not shown).

### CRISPR–Cas9-mediated mutagenesis in *G. mellonella*

Although PiggyBac transgenesis is a useful tool, we sought to further extend the molecular engineering capabilities of *G. mellonella* by testing the efficacy of CRISPR–Cas9, with respect to gene knockouts. An injection mix consisting of a KCl-buffered ribonucleoprotein complex of single sgRNA (in molar excess) targeting the eGFP sequence^44^ and mCherry-tagged Cas9 was injected into embryos homozygous for the *Bm*hsp90:GFP/3xP3:DsRed transgenic cassette. A hatch rate of 31% was observed, similar to that observed for PiggyBac injections, yet survival to pupation was low at only 7% (Table 1).

**Fig. 4:**
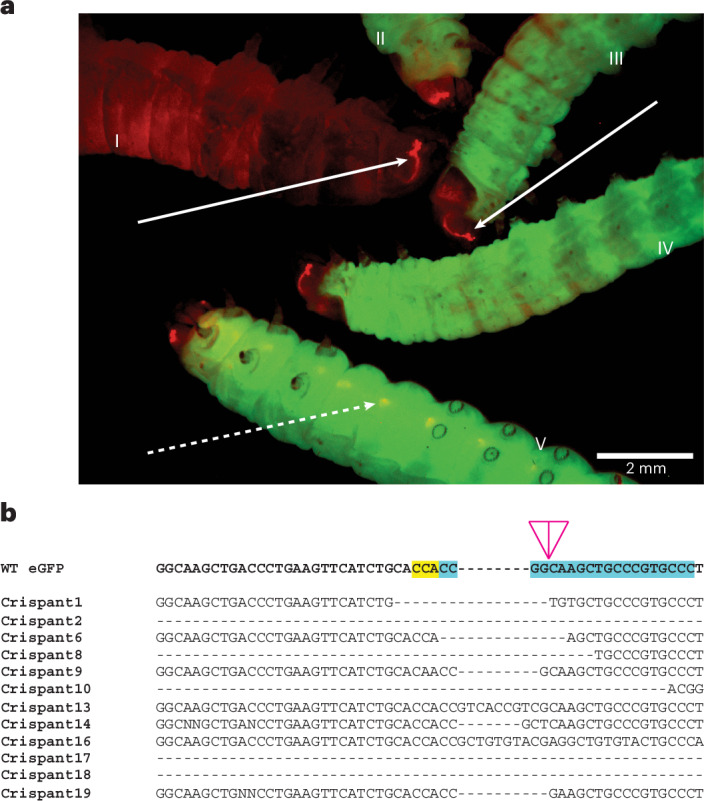
Demonstration of CRISPR in *G. mellonella.* **a**, *Bm*hsp90:GFP/3xP3:DsRed G0 larva I–V have an expression cassette inserted into their genome resulting in the expression of eGFP visible in somatic unmelanized tissue and the expression of dsRed in both eye (solid arrow) and neural (dashed arrow) tissue. Larva I is the G1 offspring of individuals of the same transgenic line as larvae II–V, but injected with a Cas9/sgRNA ribonucleoprotein targeting the eGFP gene, causing a heritable loss of function in the eGFP gene, but retaining the eye-specific dsRed expression. Larvae II–V are of the parent strain and were not injected with Cas9/sgRNA. **b**, Mutations within the coding sequences of 12 different GFP-negative G1 individuals. The G1 larvae are offspring from individuals crosses of *Bm*hsp90:GFP/3xP3:DsRed G0s injected with an sgRNA targeting the N terminus of the eGFP sequence and crossed to WT mates. The top line is the native eGFP sequence, with the sequence complementary to the sgRNA highlighted in green and the PAM sequence highlighted in red.

Among the developing G0 larval offspring, a range of eGFP knockout phenotypes was observed, ranging from minor fluorescence mosaicism to an almost complete absence of GFP fluorescence in somatic tissues (not shown). All G0 adults were outcrossed to WT mates, and the resulting broods were screened for eGFP-negative G1 larvae that were positive for DsRed expression in their stemmata (Fig. 4a). A total of 50% (14/28) of broods contained such knockout larvae, including offspring from G0 parents that had shown no, or very minor, loss of GFP expression. Analysis of the resulting CRISPR mutants via Sanger sequencing revealed a combination of small indels and larger deletions around the guide target site (Fig. 4b). Off-target effects were not screened for; instead, potential crispants (CRISPR-modified G0s individuals) and their progeny were outcrossed to WT strains for three generations before creating a stable line, thus minimizing accumulated mutations.

## Discussion

The development of advanced genetic tools for model organisms such as rodents, *Drosophila melanogaster* and zebrafish has revolutionized our ability to understand and interrogate their biology, shedding light on fundamental processes such as development, physiology and their response to environmental perturbations. Moreover, the capacity to genetically engineer these organisms to mimic human disease phenotypes has provided invaluable insights into the modes of action and potential efficacy of new therapeutic interventions. In this context, the establishment of methods for genetic modification of *G. mellonella* represents a transformative advancement with potentially profound implications for biomedical research.

Here, we have demonstrated the feasibility of both PiggyBac-mediated transformation for gene tagging and expression, as well as gene knockout using CRISPR–Cas9 technology. Both techniques rely on the injection of exogenous nucleic acid into *G. mellonella* embryos during the initial syncytial stage of development. Analysis of G0 pupae from multiple experiments using different PiggyBac constructs demonstrates that the proportions of embryos hatching, progressing to pupation and reaching eclosion vary between experiments and constructs, highlighting areas for potential improvement.

As it currently stands, PiggyBac transformation efficiencies remain low compared with those seen in other insects; in Lepidoptera, Tamura et al.^45^ reported efficiencies of 1.5% and 2.0%, versus the maximum of 0.1% efficiency that we were able to achieve (number of G1-positive broods versus number of injected eggs). We have also observed that different promoter–transposase constructs can have large effects on the transposition frequency. It is likely that further optimization of our injection methodology and helper constructs will result in increased rates of transformation. Notably, further direct comparisons between both promoters and transposases are needed to elucidate the extent to which either element is hindering integration efficiency. Exploration of DNA constructs with higher maternal or embryonic promoter activity, *G. mellonella*-specific codon optimization or the co-injection of transposase mRNA could boost integration at an earlier stage, resulting in increased transmission to the *G. mellonella* germline. In addition, the spherical nature of *G. mellonella* embryos and the absence of phenotypic markers for the anterior–posterior axis precludes targeted microinjection to the germline precursor/pole cell region, an approach that has proved valuable in enhancing transgenesis efficiency in insects such as *B. mori*^45^. Generating a transgenic *G. mellonella* line where the anterior–posterior pole of fertilized oocytes is highlighted (for example, through fluorescently tagged determinants, such as Nanos) could overcome this limitation, although a Nanos promoter/terminator construct described by Heryanto et al.^46^ did not localize mScarlet to the presumptive germ region^47^. Nonetheless, the PiggyBac donor integration rate using the methodology described here is generally at, or above, ~1 % (Table 1), making the technique as it currently stands useful to researchers seeking to adopt genetically modified *G. mellonella* as a model. The use of inverse PCR failed to generate distinct products for sequencing; as such, we were unable to identify two of the three PiggyBac insertion sites. This could potentially be due to issues with priming in the reactions or large numbers of products produced due to the regularity of HpaII sites. Alternative methods such as splinkerette PCR or whole-genome sequencing approaches could produce more reliable results in the future.

The application of CRISPR–Cas9-mediated mutagenesis, now established in various insect species, represents another substantial advancement. CRISPR–Cas9 offers a robust alternative to RNA interference, which has been shown to work in *G. mellonella*^48,49^, but with variable efficiency in other lepidopterans, possibly due to compensatory gene upregulation^50–52^. Again, although our current efficiencies are low, with further optimization of ribonucleoprotein concentrations and injection timing, survival rates and mutagenesis efficacy in *G. mellonella* could potentially reach levels comparable to those reported in *D. melanogaster*, *Tribolium castaneum*, *B. mori* and other Lepidoptera.

Given that *G. mellonella* is predominantly used as a model to understand microbial infection and host–pathogen interactions, the methods described in this study have the potential to considerably enhance its utility and adoption. While this work focuses on the generation of reporter lines to visualize subcellular structures (the microtubule cytoskeleton and nuclei), future applications could include reporters of larval health status. Examples include reporters driving fluorescence throughout the larvae, or in particular cell subtypes, upon infection by particular pathogens or upon systemic release of antimicrobial peptides. Such ‘sensor’ moth larvae would provide a quantifiable readout of health, complementary to current observation of melanisation. The ability to use CRISPR–Cas9 to knock out individual genes or entire gene families, or replace them with humanized disease variants, could also provide a bank of knockout lines that more accurately represent phenotypic traits found in human diseases, facilitating the screening of new therapeutics or interventions. Moreover, the ability to engineer *G. mellonella* not only broadens the scope of research and versatility of this model but also aligns with the principles of the 3Rs (Replacement, Reduction and Refinement). By offering a viable alternative or complementary system to rodent models, uptake of *G. mellonella* could lead to a reduction in the use of mammalian models in infection research and beyond, thus positively addressing both ethical and cost considerations of robust scientific animal research.

## Methods

### Animals and rearing

Ethical approval was sought and approved by the University of Exeter ethics review panel, and project approval was granted by the genetically modified organisms project panel. A completed ARRIVE guidelines checklist is included online (Supplementary Data 1).

An inbred WT *G. mellonella* colony at the *Galleria mellonella* Research Centre (GMRC) was reared on an artificial honey diet (Diet 3^53^) at 30 °C, constant darkness. Originally derived from commercially bred UK larvae, the GMRC colony has been continuously bred at the University of Exeter as an isolated colony since 2016. A more detailed rearing protocol is described in ref. ^41^.

Transgenic strains were reared at 30 °C on the same diet in large polypropylene fly vials (10 cm × 5 cm, Darwin Biological) with foam bungs to prevent L1 larval escape. At the late larval wandering stage, larvae were transferred to Petri dishes containing diet and allowed to pupate. Pupae were transferred to small polyethylene terephthalate jars with 5-μm wire mesh lids, and the adults were allowed to oviposit on egg papers.

### Fixation and immunostaining

Jars of WT adults were kept at 30 °C in constant darkness and allowed to lay on egg papers overnight. The egg papers were removed, and the embryos discarded before replacing the clean egg papers into the jars. The moths were allowed to oviposit undisturbed for 1 h in darkness, before the papers were again removed and the embryos collected and labeled as 0–1 h old. Embryos were allowed to develop for set time periods before dechorionating with agitation for 2 min in a diluted solution of thin bleach (1.25% active chlorine) and 0.05% Triton X-100.

Aged embryos were transferred into a 1.5-ml Eppendorf tube containing 500 μl of heptane and 500 μl of methanol, inverted several times until the majority of the embryos at the interphase of the two solutions dropped into the methanol. The heptane and any embryos remaining at the interphase were removed using a Pasteur pipette, before washing twice in fresh methanol. Embryos were stored at 4 °C for no more than a week, until use.

Embryos were rehydrated sequentially for 15 min each in 75:25 and 50:50 methanol:PBS + 0.01 % Triton X-100 (PBST), before further 15-min rehydration in PBST. They were stained with 0.5 mg/ml Hoechst 33258 in PBST for 20 min at room temperature, followed by three 5‑min washes in PBST.

Larval tissues were fixed in 4% paraformaldehyde/PBS + 0.1% Tween for 1 h, stained overnight with a rabbit anti-GFP polyclonal antibody (Abcam 6556) and labeled using an appropriate Alexa Fluor 488 secondary dye (Molecular Probes).

### Imaging

Embryos were mounted on microscope slides between two stacked ring binder reinforcement stickers in Vectashield mounting medium (VectorLabs) and imaged using a Nikon TE-2000U inverted microscope. Larvae were anesthetized using CO_2_ and imaged under a Leica MZ10F fluorescence stereomicroscope with a GXCAM HiChrom-HR4 HiSens camera. Fixed larval tissues were imaged using a Leica SP-8 confocal microscope.

### RNA/DNA extraction and PCR

*Galleria mellonella* tissue samples were flash frozen in liquid nitrogen and homogenized in Eppendorf tubes using disposable microcentrifuge pestles (DWK Life Sciences), then stored on ice until use.

Total RNA was extracted from homogenates of 0–6 h embryos and the posterior third of larvae (larval end segment) using a TRIzol reagent/chloroform extraction according to the manufacturer’s protocol, and ethanol-precipitated before either immediate use or storage at –80 °C. cDNA was generated using a High-Capacity cDNA reverse transcription kit (Applied Biosystems) using the manufacturer’s protocol.

Genomic DNA was extracted from whole or partial tissue samples, depending on the size, using the New England Biolabs (NEB) gDNA extraction kit with NEB’s insect tissue protocol.

DNA fragments for diagnostic purposes were amplified using a GoTaq Hot Start mastermix (Promega). For all other purposes (including sequencing), fragments were amplified using a KOD Hot Start polymerase kit (Toboyo), according to the manufacturer’s instructions.

### Plasmids

Plasmids pHA3PIG^54^ and pBAChsp90GFP-3xP3DsRed^42^ were a kind gift from Professor Hideki Sezutsu. Henceforth, we will refer to pBAChsp90GFP-3xP3DsRed as p*Bm*hsp90:GFP/3xP3:DsRed to differentiate between the different *G. mellonella* and *B. mori* hsp90 promoters. All plasmids were assembled using Gibson assembly.

p*Bm*hsp90:hyPB was generated by inserting the *B.* mori *hsp90* 2.9-kb fragment from p*Bm*hsp90:GFP/3xP3:DsRed and the hyper-active Piggybac transposase from SPB-DNA (Hera Biolabs) upstream of WT PiggyBac transposase 3′ untranslated region from pHA3PIG in the digested backbone of pHA3PIG.

p*Gm*hsp90:GFP-αtub1b was generated by creation of an expression cassette consisting of the 2-kb upstream region of *G. mellonella hsp83* (*hsp90*) placed directly upstream of an N-terminal eGFP-tagged *G. mellonella* α-tubulin 1b cDNA sequence (LOC113521067) and SV40 polyA terminator, which was inserted between the two PiggyBac inverted terminal repeats of the digested p*Bm*hsp90:GFP/3xP3:DsRed backbone.

p*Bm*hsp90:histone2av-mCh/3xP3:DsRed was generated by digesting the p*Bm*hsp90:GFP/3xP3:DsRed plasmid with PmlI and AscI and inserting a synthesized fragment consisting of the *G. mellonella* Histone 2AV sequence (LOC113518755) connected to mCherry, via a short linker with a SV40 polyA terminator.

All plasmids were propagated in NEB 10β cells and midi-prepped using a Nucleobond Xtra midi kit (Macherey-Nagel). They were ethanol precipitated before reconstituting in either nuclease-free water or filter-sterilized 5 mM phosphate/5 mM KCl injection buffer, pH 7.4. Annotated plasmid sequences and maps are available in Supplementary Datasets 1–7 and Supplementary Figs. 3–9.

### Embryo microinjection

The injection protocol is described in detail in ref. ^41^. In brief, embryos were collected from communal adult jars containing 75 adults of mixed sex and allowed to age for 1–2 h at 30 °C, before being dechorionated with a diluted bleach solution, aligned along the edge of coverslips and glued to glass slides.

Injection mixes were made by adding plasmids, guides or proteins sequentially to the injection buffer and checking total concentration using a Nanodrop. Before use, injection mixes were spun at 16,000*g* for 1 min, before gently aspirating off the upper 90% and transferring it to a fresh Eppendorf tube. The spun mixes were then stored on ice until use.

Embryos were injected using an Eppendorf Injectman 4 microinjection system mounted to a Nikon Eclipse TE2000-U inverted microscope with a small volume of injection mix, equal to a droplet roughly 1/5 diameter of the embryo. For Piggybac-mediated mutagenesis, embryos were injected between 3 h and 5 h PO, and the injection droplet was placed on the side of the embryo relative to the putative anterior–posterior poles. For CRISPR–Cas9, embryos were injected at 2.0–3.5 h PO and injection droplets were placed in the center of the embryo.

### Post-injection rearing and screening

After injection, embryos were reared as described in ref. ^41^. Late-stage G0 larvae larvae were removed from the diet and screened for visible changes in somatic fluorescence using a fluorescence stereomicroscope (Leica MZ10F or Olympus SZX-16). Those demonstrating mosaic fluorescence were separated into fresh diet. Once pupated, putative transformants were sexed based on genital morphology visible in the terminal segments, and G0 adults were mated either to a mixture of siblings and WTs (for PiggyBac-injected G0s) or WTs only (for CRISPR–Cas9-injected G0s). Embryos were collected from each cross, and the progeny were screened for visible changes in somatic fluorescence at both embryonic and larval stages. Stable transgenic lines were generated by selecting the G1 generation with the brightest phenotype and outcrossing for two to three further generations, before sibling mating and screening for the brightest offspring. These were then sibling mated and screened for consistent phenotype over multiple generations.

### Guide RNA synthesis and Cas9

An anti-eGFP sgRNA previously described by Jao et al.^44^ was synthesized in vitro, as described in Burger et al.^55^. A DNA template for the sgRNA was generated by PCR amplification using two primers (sgRNA-EGFP forward and a PAGE-purified sgRNA reverse), followed by purification of the PCR product (Promega). In vitro transcription was performed overnight at 37 °C using a T6 RNA polymerase (Roche), followed by DNase treatment, RNA cleanup and validation for size and presence of a single band on a denaturing MOPS–formaldehyde gel. The sgRNA was stored at −80 °C until use.

The Cas9 protein used was a kind gift from Professor Christian Mosimann and is a modified version of *S. pyogenes* Cas9 fused in frame with an additional C-terminal HA tag, a bipartite nuclear location sequence, an mCherry polypeptide sequence and an additional monopartite nuclear location sequence at the C terminus after the mCherry sequence^55^.

### Mutation analysis and sequencing

Piggybac insertion sites were verified via inverse PCR. gDNA from G1 transgenic larvae was digested for 2 h using HpaII, followed by heat inactivation. Genomic fragments were then self-ligated using T4 DNA ligase at 4 °C overnight, followed by ethanol precipitating. The genomic regions surrounding the Piggybac entry sites were amplified through PCR using two sets of primers specific to the 5′ and 3′ Piggybac inverted tandem repeats (ITRs) (iPCR 5′ F & R and iPCR 3′ F & R), before sequencing using a commercial short read service (Eurofins). Mutations in the GFP coding region were amplified using primers for GFP (GFPF and GFPR) and sequenced using a commercial short read service (Eurofins). All primers all listed in Supplementary Table 1.

### Reporting summary

Further information on research design is available in the Nature Portfolio Reporting Summary linked to this article.

## Online content

Any methods, additional references, Nature Portfolio reporting summaries, source data, extended data, supplementary information, acknowledgements, peer review information; details of author contributions and competing interests; and statements of data and code availability are available at 10.1038/s41684-025-01665-7.

## Supplementary information

Supplementary Information Supplementary Figs. 1–9 and Table 1.

Reporting Summary

Supplementary Data 1 ARRIVE guidelines.

Supplementary Dataset 1 pHA3PIG.

Supplementary Dataset 2 p*Bm*hsp90:hyPBase.

Supplementary Dataset 3 p*Bm*hsp90:GFP/3xP3:DsRed.

Supplementary Dataset 4 p*Gm*hsp90:GFP.

Supplementary Dataset 5 p*Gm*hsp90:GFP-αtub1b.

Supplementary Dataset 6 p*Bm*hsp90:histone2av-mCh/3xP3:DsRed.

Supplementary Dataset 7 *Bm*hsp90:GFP/3xP3:DsRed transposon insertion chr12 sequence.

## Data Availability

Raw data were generated at the University of Exeter Sequencing service and the University of Exeter Bioimaging Facility. Derived data supporting the findings of this study are available from the corresponding authors on request.
