# Supplementary material for: PiggyBac-mediated transgenesis and CRISPR–Cas9 knockout in the greater wax moth, Galleria mellonella
Source: Lab Anim (NY). 2026 Feb 10;55(3):95–102. doi: 10.1038/s41684-025-01665-7 (PMC12956550; doi:10.1038/s41684-025-01665-7)
Supplement: Supplementary file 1 — Supplementary Figs. 1–9 and Table 1. [file 41684_2025_1665_MOESM1_ESM.pdf]

lab animal

https://doi.org/10.1038/s41684-025-01665-7

PiggyBac-mediated transgenesis and CRISPR–Cas9 knockout in the greater wax moth, *Galleria mellonella*

In the format provided by the authors and unedited

Supplemental Figure 1: *Galleria mellonella* embryos fixed 12-14hrs into development and stained with Hoechst 33258 DNA dye.

Embryos at both 12-13 and 13-14 hours post ovipostition can now be seen to have areas with higher density of nuclei vs others. We hypothesise that the embryonic layer consists of the more densely nucleated layer which will form the germ band and the less densely nucleated extraembryonic region will form the serosa.

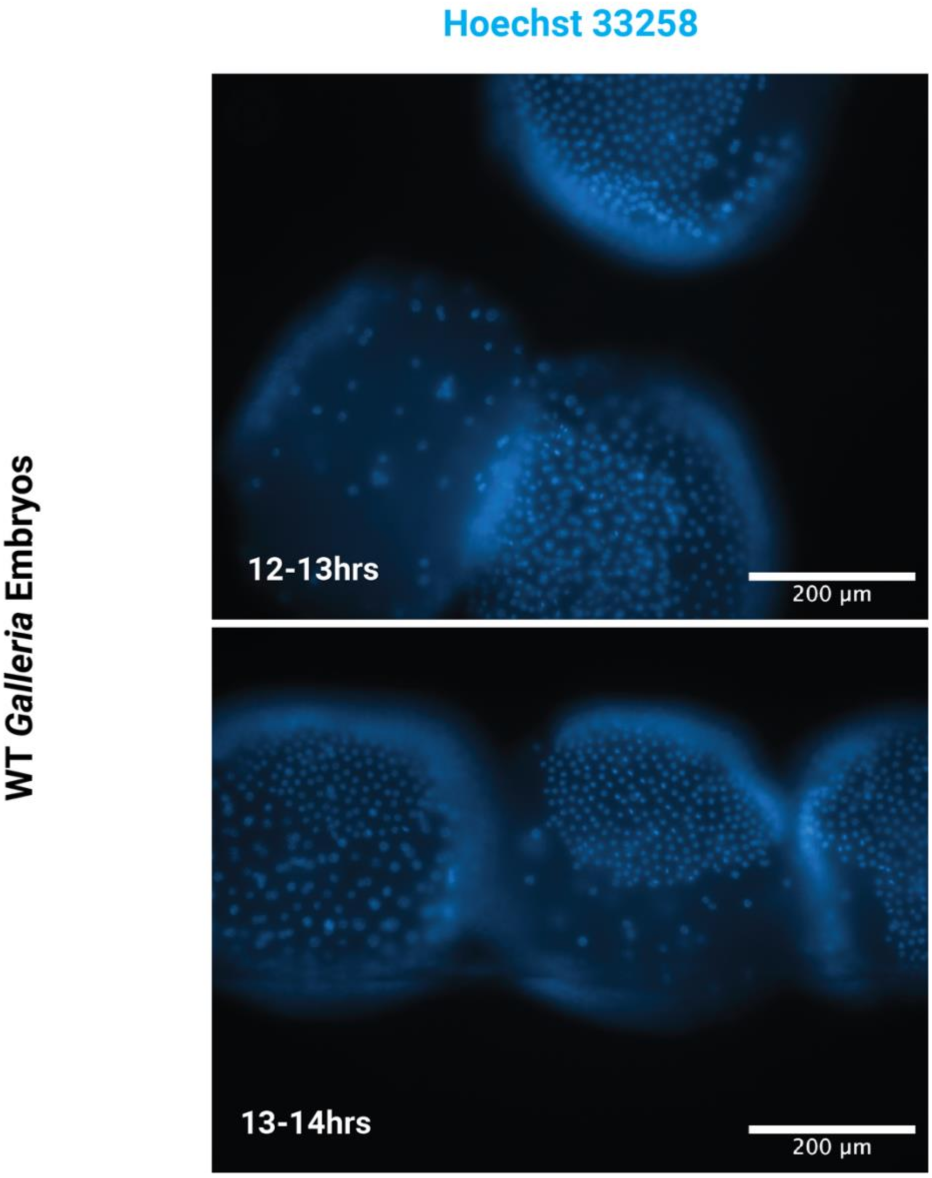

Supplemental Figure 2: PCR confirmation of *Bm*shp90:GFP/3xP3:DsRed insertion locus

PCR amplification across the suspected insertion locus of *Bm*shp90:GFP/3xP3:DsRed using primers GFP P3 iPCR confirm F and R (Supplemental table 1) reveal a 400bp band present in gDNA from wild type larvae which is then absent in 5 transgenic larvae.

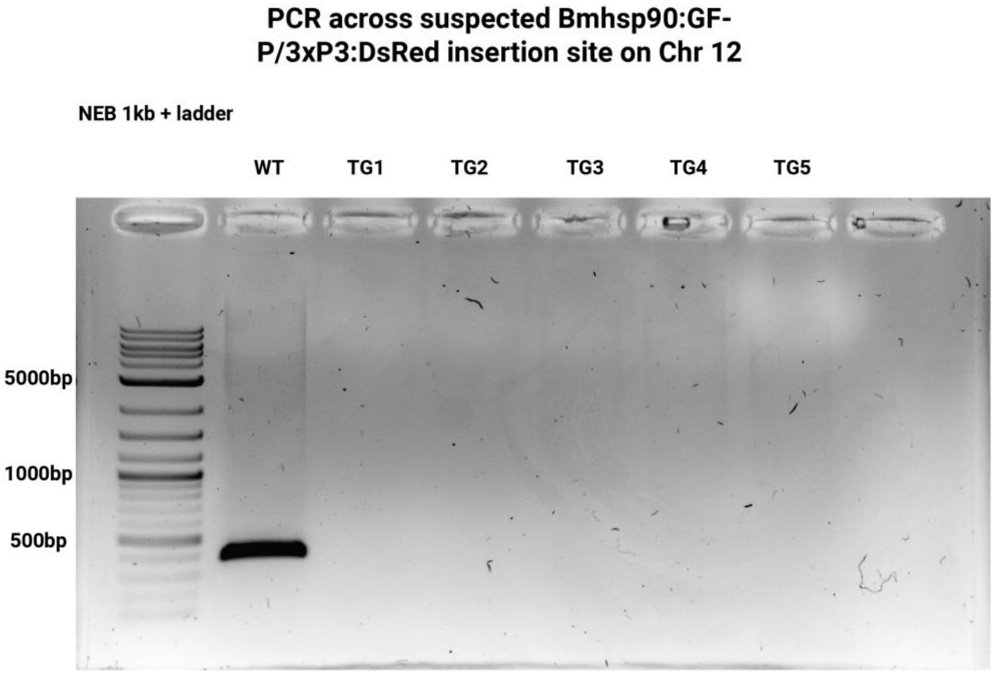

Supplemental Figure 3. Plasmid map of pHA3PIG

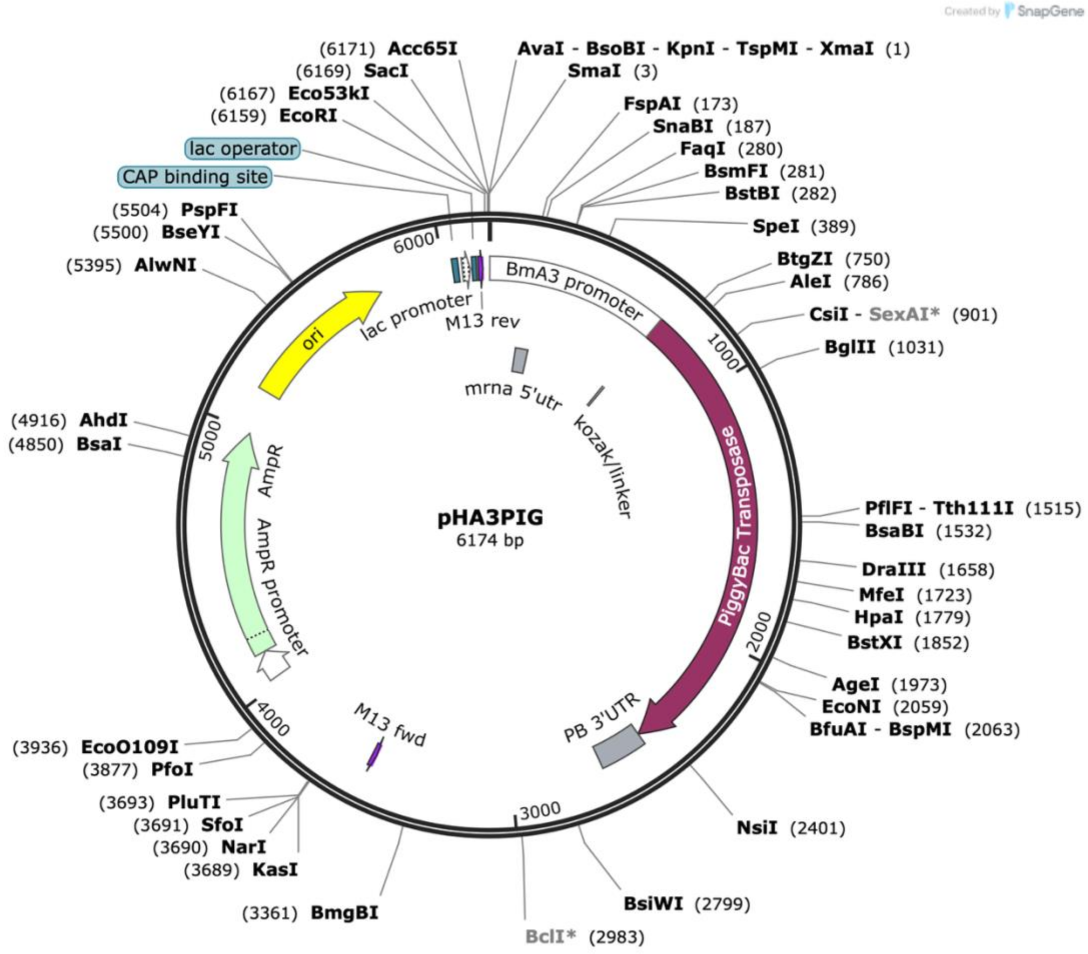

Supplemental Figure 4. Plasmid map of pBmhsp90(hyPBase)

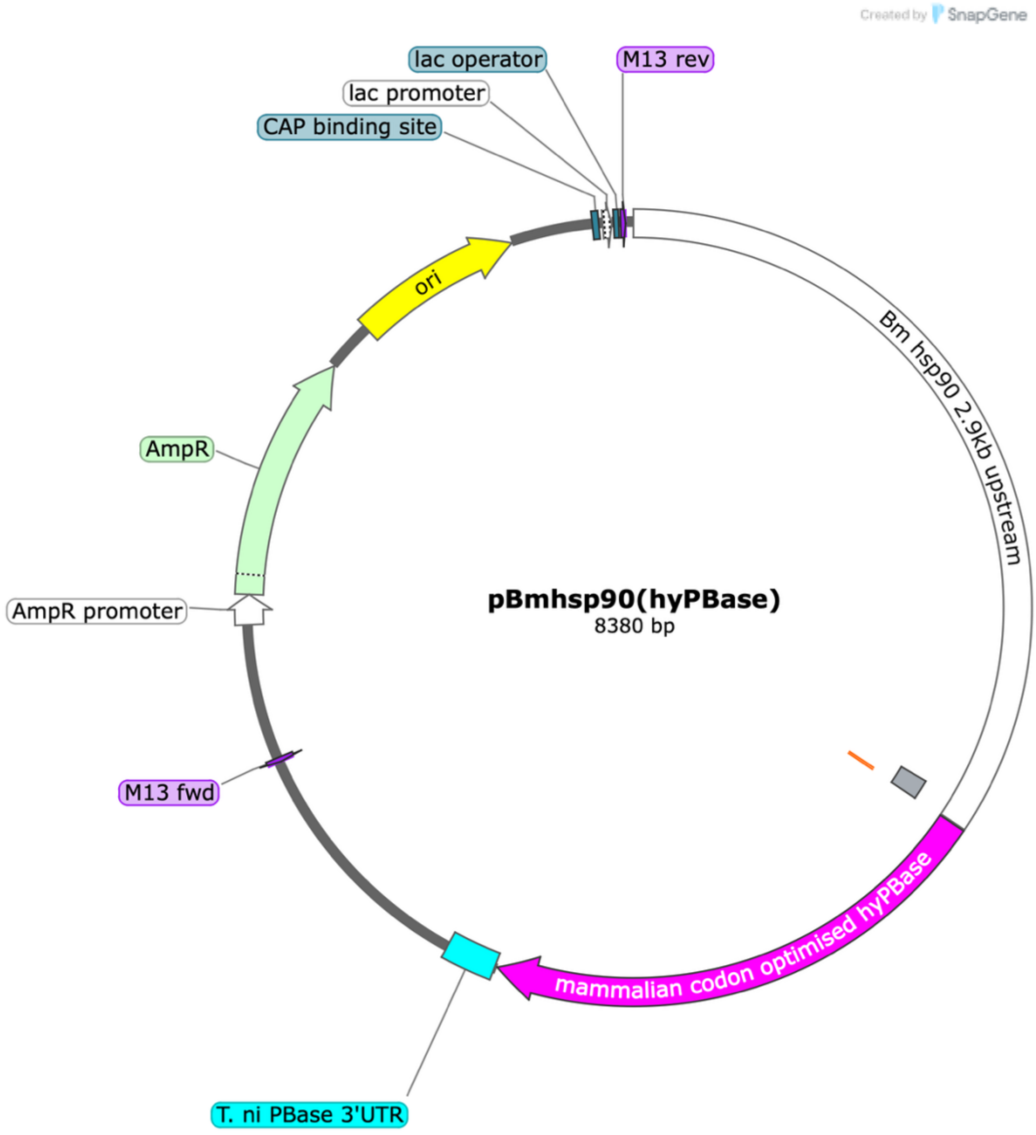

Supplemental Figure 5. Plasmid map of pBmhsp90(GFP)3xP3(DsRed)

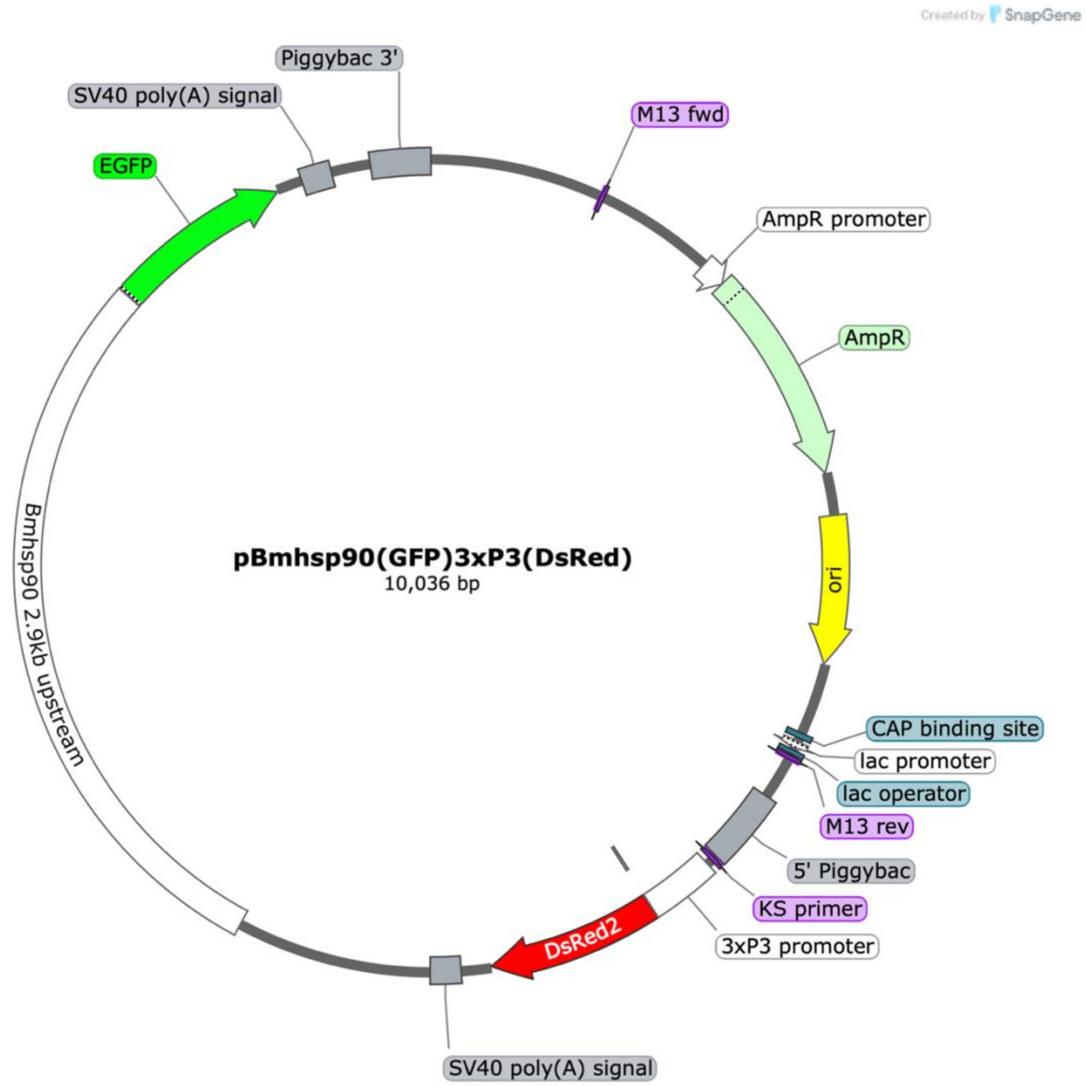

Supplemental Figure 6. Plasmid map of pHA3PIG

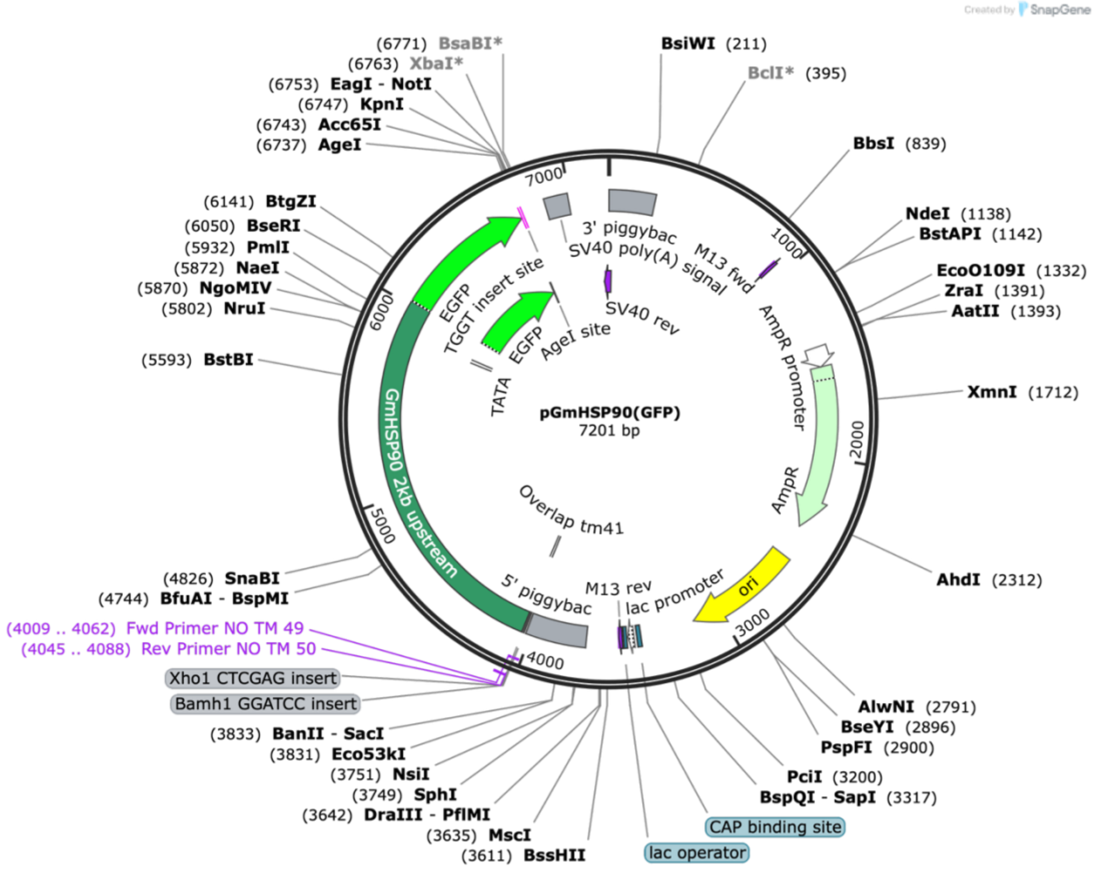

Supplemental Figure 7. Plasmid map of pGmhsp90(GFP-alphatub1b)

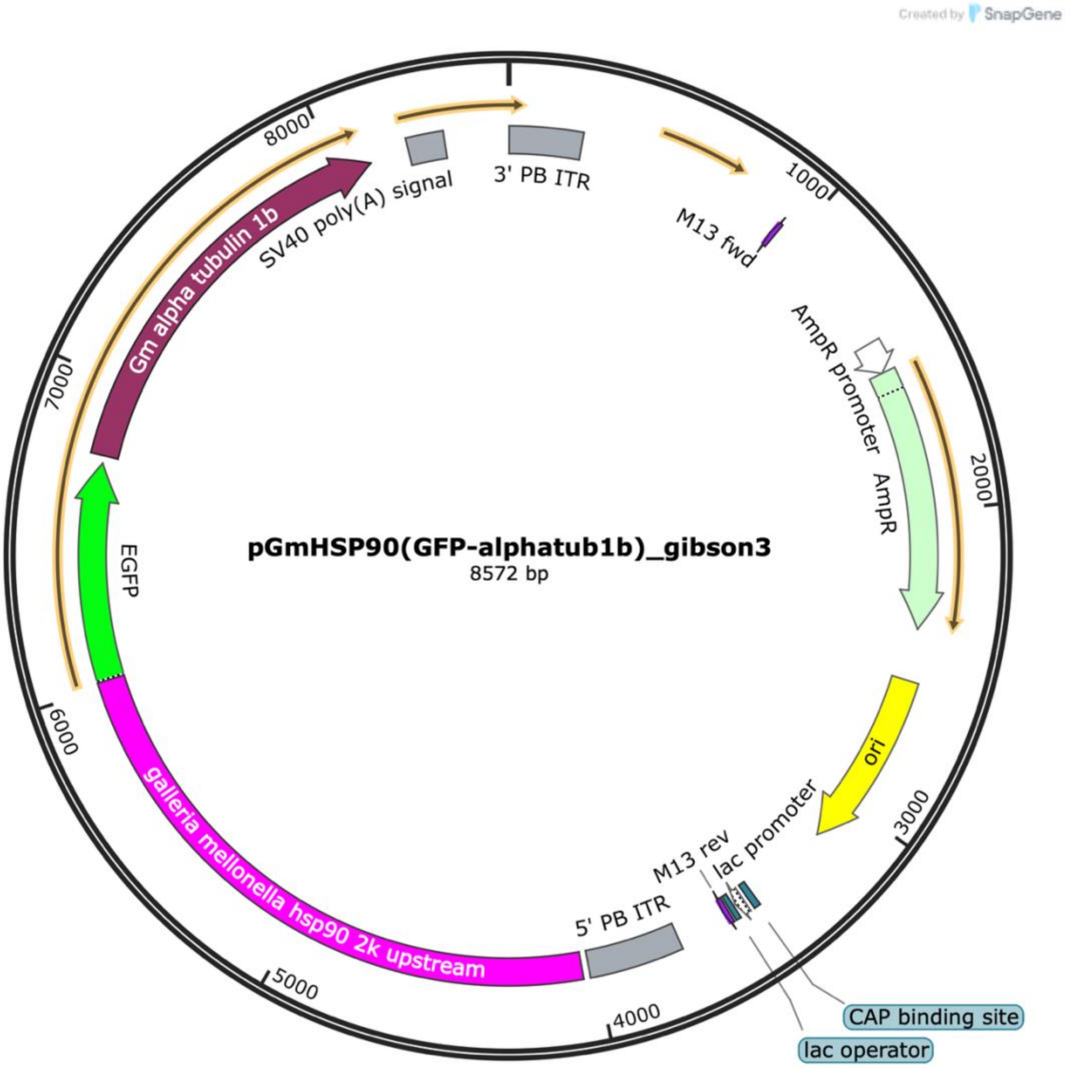

Supplemental Figure 8. Plasmid map of pBmhsp90(histone2av-mCh)3xP3(DsRed)

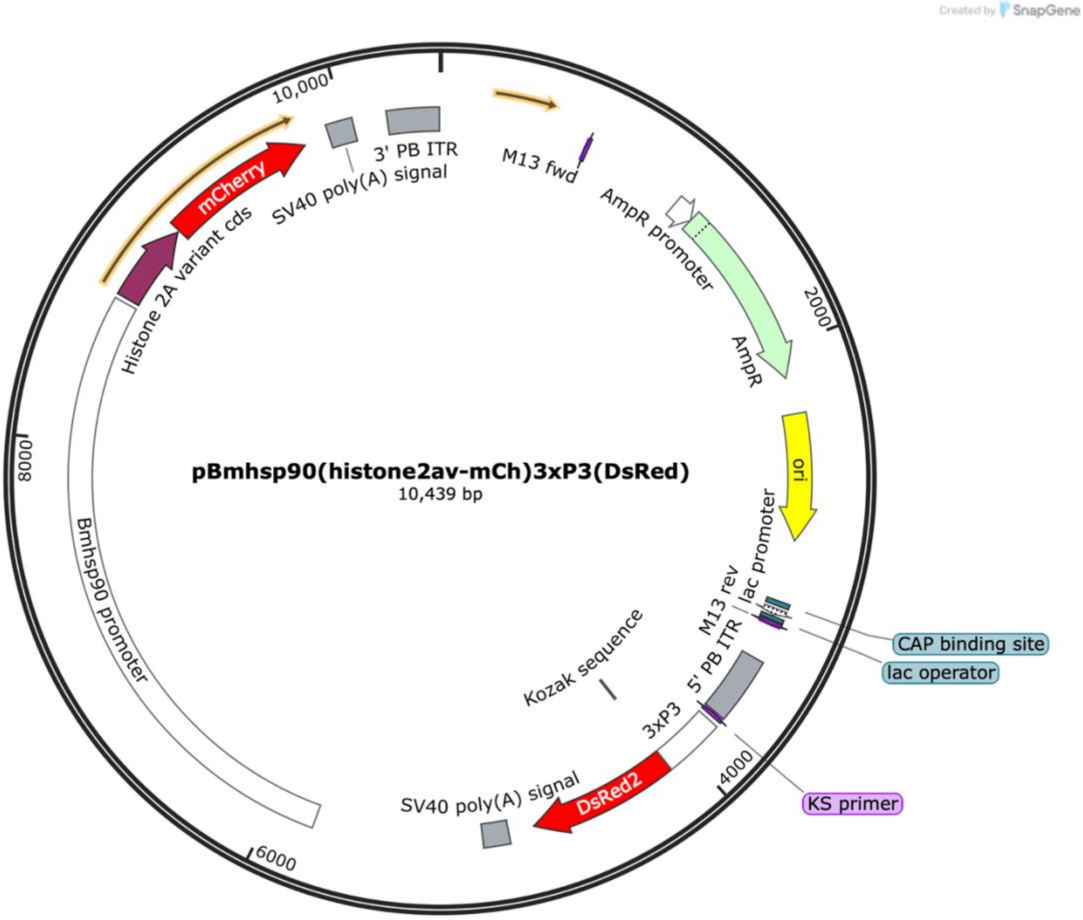

Supplemental Figure 9. Gene map of Bmhsp90(GFP)3xP3(dsRed) transposon insertion chr12

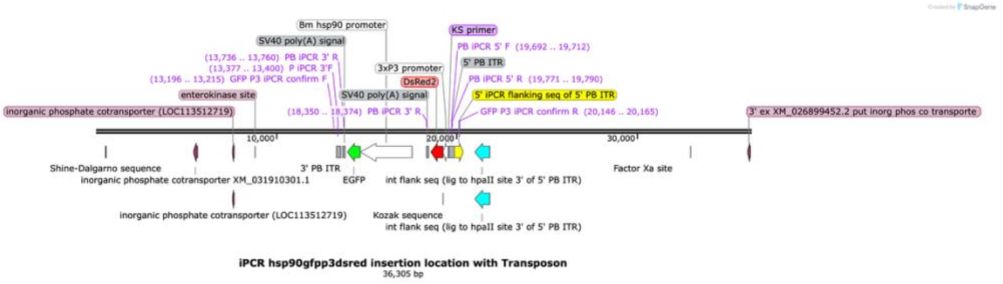

Supplemental Table 1: Primers described for generation of Gibson constructs, iPCR and sequencing

Primer set	Name	Seq
**p*Bm*hsp90:hyPB gibson primers**		
	hsp90 + op codon motif_fwd	cgaattcgagctcggtacccccatggctcagttcgctttaaatatc
	hsp90 + op codon motif_rev	tgctgcccattttgataattcacacaaaatgactagaggg
	mamm hyPBase_fwd	aattatcaaaatgggcagcagcctggac
	mamm hyPBase_rev	tattagtcagtcagaaacagctctggcacatg
	3'UTR + kasI_fwd	ctgtttctgactgactaataagtataatttgtttctattatgtataagttaagctaattac
	3'UTR + kasI_rev	aggagaaaataccgcatcaggcgccattcgccattcag
**p*Gm*hsp90:GFP gibson primers**		
	Gmel hsp90 2kb upstream_fwd	gcttatcgataccgtcgaccgtacctattttagaatacacggattcataatatataatatacc
	Gmel hsp90 2kb upstream_rev	tgctcaccatcttggttatttcacaattaaatgtaatactggtttaac
	EGFP + TGGT linker_fwd	aataaccaagatggtgagcaagggcgag
	EGFP + TGGT linker_rev	tcgcggccgcttaggtaccaccggtcttg
	SV40 w/ NotI site_fwd	tggtacctaagcggccgcgactctagatc
	SV40 w/ NotI site_rev	aagtaacaaaacttttatggcatagatttcatcttcattcgtgtcacgaaatg
**p*Gm*hsp90:GFP-αtub1b gibson primers**		
	a tub 1b gibson _fwd	ctgtacaagaccggtggtaccggcgcgcctatgcgtgagtgcatctcc
	a tub 1b gibson _rev	ttaatattcttctgccccctc
**iPCR**		
	iPCR 5'_Fwd	accgcgtgagtcaaaatgacg
	iPCR 5'_Rev	ccaagcggcgactgagatgt
	iPCR 3'_Fwd	cagaccgataaaacacatgcgtca
	iPCR 3'_Rev	tggacaaaccacaactagaatgcag
**Chromosome 12 insertion confirmation**		
	GFP P3 iPCR confirm F	gacggggcaccttggtatag
	GFP P3 iPCR confirm R	agacgcttttgcttggagga
**eGFP sgRNA**		
	eGFP sgRNA fwd	gaaattaatacgactcactatagggcacgggcagcttgccgggttttagagctagaaatagc
	sgRNA rev	aaaagcaccgactcggtgccactttttcaagttgataacggactagccttattttaacttgctatttctagctctaaaac
**eGFP primers**		
	GFP_Fwd	ctggtcgagctggacggcgacg
	GFP_Rev	cacgaactccagcaggaccatg
