# Supplementary material for: PiggyBac-mediated transgenesis and CRISPR–Cas9 knockout in the greater wax moth, Galleria mellonella
Source: Lab Anim (NY). 2026 Feb 10;55(3):95–102. doi: 10.1038/s41684-025-01665-7 (PMC12956550; doi:10.1038/s41684-025-01665-7)
Supplement: Supplementary file 3 — ARRIVE guidelines. [file 41684_2025_1665_MOESM3_ESM.pdf]

NOTE: Please save this file locally before filling in the table, DO NOT work on the file within your internet browser as changes will not be saved. Adobe Acrobat Reader (available free here) is recommended for completion.

The ARRIVE guidelines 2.0: author checklist

The ARRIVE Essential 10

These items are the basic minimum to include in a manuscript. Without this information, readers and reviewers cannot assess the reliability of the findings.

Item		Recommendation	Section/line number, or reason for not reporting
**Study design**	1	For each experiment, provide brief details of study design including:<br>a. The groups being compared, including control groups. If no control group has been used, the rationale should be stated.<br>b. The experimental unit (e.g. a single animal, litter, or cage of animals).	Most experiments in this article sought to investigate whether a technique was feasible, and thus did not require comparison or a control group. Where a direct comparison was sought, in using new helper plasmids, wild type embryos injected [illegible]<br>the experimental unit here was a single embryo
**Sample size**	2	a. Specify the exact number of experimental units allocated to each group, and the total number in each experiment. Also indicate the total number of animals used.<br>b. Explain how the sample size was decided. Provide details of any *a priori* sample size calculation, if done.	Table 1<br>sample size was estimated based on microinjection survival rate, however calculations were severely limited due to no prior information on transgenesis efficienc[illegible]
**Inclusion and exclusion criteria**	3	a. Describe any criteria used for including and excluding animals (or experimental units) during the experiment, and data points during the analysis. Specify if these criteria were established *a priori*. If no criteria were set, state this explicitly.<br>b. For each experimental group, report any animals, experimental units or data points not included in the analysis and explain why. If there were no exclusions, state so.<br>c. For each analysis, report the exact value of *n* in each experimental group.	no criteria were set<br>no exclusions were set<br>n/a
**Randomisation**	4	a. State whether randomisation was used to allocate experimental units to control and treatment groups. If done, provide the method used to generate the randomisation sequence.<br>b. Describe the strategy used to minimise potential confounders such as the order of treatments and measurements, or animal/cage location. If confounders were not controlled, state this explicitly.	none<br>where comparisons were drawn injections were performed within an hour of each other using embryos from the same batch of eggs.
**Blinding**	5	Describe who was aware of the group allocation at the different stages of the experiment (during the allocation, the conduct of the experiment, the outcome assessment, and the data analysis).	experiments were not blinded
**Outcome measures**	6	a. Clearly define all outcome measures assessed (e.g. cell death, molecular markers, or behavioural changes).<br>b. For hypothesis-testing studies, specify the primary outcome measure, i.e. the outcome measure that was used to determine the sample size.	Table 1<br>presence or absence of integration of transgenic construct (visible fluorescence)
**Statistical methods**	7	a. Provide details of the statistical methods used for each analysis, including software used.<br>b. Describe any methods used to assess whether the data met the assumptions of the statistical approach, and what was done if the assumptions were not met.	n/a<br>n/a
**Experimental animals**	8	a. Provide species-appropriate details of the animals used, including species, strain and substrain, sex, age or developmental stage, and, if relevant, weight.<br>b. Provide further relevant information on the provenance of animals, health/immune status, genetic modification status, genotype, and any previous procedures.	line 290-300<br>wild type embryos were used, apart from for CRISPR in which ones expressing a transgenic eGFP/DsRed2 dual cassette were used (Line 193-212)
**Experimental procedures**	9	For each experimental group, including controls, describe the procedures in enough detail to allow others to replicate them, including:<br>a. What was done, how it was done and what was used.<br>b. When and how often.<br>c. Where (including detail of any acclimatisation periods).<br>d. Why (provide rationale for procedures).	microinjection covered in a separate article (ref 41) , rest of methods covered 288-423<br>timings for development covered 74-93<br>n/a<br>microinjection necessary for introduction of foreign DNA/RNA/protein into embryo
**Results**	10	For each experiment conducted, including independent replications, report:<br>a. Summary/descriptive statistics for each experimental group, with a measure of variability where applicable (e.g. mean and SD, or median and range).<br>b. If applicable, the effect size with a confidence interval.	n/a<br>n/a
